# Gamma sensory stimulation in mild Alzheimer's dementia: An open‐label extension study

**DOI:** 10.1002/alz.70792

**Published:** 2025-10-25

**Authors:** Diane Chan, Gabrielle de Weck, Brennan L. Jackson, Ho‐Jun Suk, Noah P. Milman, Erin Kitchener, Vanesa S. Fernandez Avalos, M. J. Quay, Kenji Aoki, Erika Ruiz, Andrew Becker, Monica Zheng, Remi Philips, Rosalind Firenze, Ute Geigenmüller, Bruno Hammerschlag, Steven Arnold, Pia Kivisäkk, Michael Brickhouse, Alexandra Touroutoglou, Emery N. Brown, Edward S. Boyden, Bradford C. Dickerson, Elizabeth B. Klerman, Li‐Huei Tsai

**Affiliations:** ^1^ Picower Institute for Learning and Memory, Department of Brain and Cognitive Sciences Massachusetts Institute of Technology Cambridge Massachusetts USA; ^2^ Department of Neurology Massachusetts General Hospital Boston Massachusetts USA; ^3^ Department of Neurology Harvard Medical School Boston Massachusetts USA; ^4^ Health Sciences and Technology Massachusetts Institute of Technology Cambridge Massachusetts USA; ^5^ Department of Behavioral and Systems Neuroscience Oregon Health and Sciences University Portland Oregon USA; ^6^ Brenner Center for Psychological Assessment and Consultation William James College Newton Massachusetts USA; ^7^ The Winsor School Boston Massachusetts USA; ^8^ Institute for Medical Engineering and Sciences Massachusetts Institute of Technology Cambridge Massachusetts USA; ^9^ Institute for Data Systems and Society Massachusetts Institute of Technology Cambridge Massachusetts USA; ^10^ Department of Anesthesia, Critical Care and Pain Medicine Massachusetts General Hospital Boston Massachusetts USA; ^11^ McGovern Institute, Department of Brain and Cognitive Sciences Massachusetts Institute of Technology Cambridge Massachusetts USA; ^12^ Department of Biological Engineering Massachusetts Institute of Technology Cambridge Massachusetts USA; ^13^ Center for Neurobiological Engineering Massachusetts Institute of Technology Cambridge Massachusetts USA; ^14^ Koch Institute Massachusetts Institute of Technology Cambridge Massachusetts USA; ^15^ Howard Hughes Medical Institute Cambridge Massachusetts USA; ^16^ Yang Tan Collective Massachusetts Institute of Technology Cambridge Massachusetts USA; ^17^ Division of Sleep Medicine Harvard Medical School Boston Massachusetts USA; ^18^ Broad Institute of MIT and Harvard Cambridge Massachusetts USA

**Keywords:** 40 Hz audiovisual stimulation, ADNI, Alzheimer's disease, LEADS, long‐term treatment, NACC, plasma pTau217

## Abstract

**INTRODUCTION:**

We evaluated the long‐term effects of daily 40 Hz (gamma frequency) audiovisual stimulation on cognition and biomarkers in five patients with mild Alzheimer's disease (AD).

**METHODS:**

Over 2 years, patients received 1‐h daily stimulation. Electroencephalography (EEG) was used to assess neural entrainment; magnetic resonance imaging (MRI) measured brain volumes; actigraphy monitored activity patterns; neuropsychological tests evaluated cognition; and S‐PLEX assay measured plasma pTau217.

**RESULTS:**

No adverse events occurred over the study period. Three female patients with late‐onset AD (LOAD) retained strong EEG entrainment and showed less decline in Mini‐Mental State Examination (MMSE), Clinical Dementia Rating (CDR), and Functional Assessment Scale (FAS) scores compared to matched controls from National Alzheimer's Coordinating Center (NACC), Alzheimer's Disease Neuroimaging Initiative (ADNI), and Longitudinal Early‐Onset Alzheimer's Disease Study (LEADS). Plasma samples were available for only two of five participants – both with LOAD – and both showed pTau217 reductions of 47% and 19%.

**DISCUSSION:**

These findings suggest that long‐term 40 Hz audiovisual stimulation is safe, feasible, and may offer cognitive and biomarker benefits in some individuals with mild AD, supporting further investigation.

**CLINICAL TRIAL REGISTRATION INFORMATION:**

ClinicalTrials.gov (NCT04055376).

**Highlights:**

- Five mild Alzheimer's disease (AD) patients safely used daily 40 Hz audiovisual stimulation for 2 years.
- Late‐onset AD (LOAD) patients showed increased 40 Hz electroencephalography (EEG) power and improved cognitive scores.
- National Alzheimer's Coordinating Center (NACC) data enhanced early‐phase analysis and support precision medicine in AD studies.
- Plasma pTau217 declined in 2 LOAD patients after 2 years of daily use.
- This small pilot is the first to link long‐term 40 Hz therapy to AD biomarker change.

## 1 BACKGROUND

Alzheimer's disease (AD) is a complex neurodegenerative disorder characterized by the progressive accumulation of amyloid‐beta plaques and phosphorylated tau tangles as hallmark pathological features.^1^ Beyond these molecular pathologies, disruptions in neuronal network oscillations, including gamma‐band oscillations (30–80 Hz), have been observed in AD mouse models^2^, ^3^, ^4^, ^5^ and patients with AD.^6^, ^7^ Emerging evidence indicates that evoked 40 Hz gamma oscillations using sensory stimulation – or Gamma Entrainment Using Sensory (GENUS) stimuli – may help mitigate AD pathology and support cognitive function in transgenic animal models.^8^ Using mouse models of AD and neurodegeneration, we found that visual and auditory 40 Hz light stimulation decreases amyloid levels and ameliorates cognitive impairment, with combined stimulation yielding enhanced effects.^9^, ^10^, ^11^, ^12^ These findings have since been validated and expanded upon in numerous animal models.^13^, ^14^, ^15^, ^16^, ^17^, ^18^, ^19^, ^20^, ^21^

Several clinical trials have explored the effects of gamma frequency sensory stimulation in AD patients. A feasibility study demonstrated that 1‐h daily 40 Hz sensory stimulation with GENUS is safe, well‐tolerated, and associated with reduced brain atrophy and improved sleep and memory after 3 months of use.^22^ Another trial reported improved sleep quality and activities of daily living in AD patients after 4–8 weeks of 40 Hz stimulation, suggesting potential therapeutic benefits.^23^ Additionally, a 6‐month trial in mild to moderate AD found that gamma stimulation was safe and may offer cognitive benefits and improved outcomes on the ADCS‐ADL (Alzheimer's Disease Cooperative Study – Activities of Daily Living) scale and for sleep scores.^24^ The same group also reported white matter preservation, reduced myelin loss,^25^, ^26^ and less brain atrophy^27^ after 6 months of 40 Hz light and sound stimulation, warranting further investigation. While these early findings are promising, prior trials are limited to short‐term (1–6 months) interventions. Here, we extend these findings by describing the long‐term effects of daily at‐home GENUS on cognition, biomarkers, sleep parameters, and safety in patients with mild AD dementia after approximately 2 years of daily 1‐h usage.

**RESEARCH IN CONTEXT**

1. **Systematic review**: The authors reviewed literature in PubMed about the effects of 40 Hz audiovisual stimulation in patients with mild Alzheimer's disease (AD). No study yet has investigated the safety and efficacy of stimulation for more than 6 months.
2. **Interpretation**: No adverse effects were reported by three female patients with mild late‐onset AD (LOAD) and two male patients with mild early‐onset AD (EOAD) who used 1‐h daily 40 Hz audiovisual stimulation for 2 years. 40 Hz EEG response to stimulation declined in EOAD but increased in LOAD patients, who demonstrated significantly improved Mini‐Mental State Examination (MMSE), Clinical Dementia Rating (CDR), and FAS scores compared to matched subjects from National Alzheimer's Coordinating Center (NACC), Alzheimer's Disease Neuroimaging Initiative (ADNI), and Longitudinal Early‐Onset Alzheimer's Disease Study (LEADS). Two LOAD patients showed a decrease of 47% and 19% in plasma levels of pTau217.
3. **Future directions**: These results provide the rationale for larger long‐term studies of 40 Hz audiovisual stimulation in LOAD patients and underscore the utility of nationwide AD studies such as NACC as a source of no‐treatment controls.

## 2 METHODS

Five participants from our original single‐blinded, randomized, placebo‐controlled trial (NCT04055376)^22^ that was designed to evaluate the safety and efficacy of 40 Hz GENUS in patients with mild AD dementia, chose to continue in an open‐label extension. Four of these participants[Table alz70792-tbl-0001] had been in the control arm of the original trial (CONSORT diagram, Figure S1) and switched to using 1‐h daily GENUS stimulation at month 6 (LOAD1) or month 9 (LOAD3, EOAD1, EOAD2), and one participant (LOAD2) had been included in the treatment arm and continued to use 1‐h daily GENUS stimulation for a total of 30 months from baseline. 40 Hz light and sound GENUS stimulation was delivered at home using the devices described in Chan et al. (2022), consisting of a 2′ by 2′ light emitting diode (LED) panel and a speaker delivering synchronized 40 Hz light and sound, with a centrally mounted tablet to provide entertainment (Figure S2). Scalp electroencephalography (EEG), actigraphy, T1‐weighted magnetic resonance imaging (MRI), and neuropsychological testing were performed and analyzed essentially as described before,^22^ except that Freesurfer version 7.4.1 was used for MRI analysis and MNE‐Python^28^ for EEG analysis. All measurements were taken at time points, including at least 0, 3, and ∼30 months, relative to the start of the original trial. Adverse events were recorded on structured questionnaires. Average changes in test scores or brain‐structure volumes were estimated and corrected for confounding variables using the scikit‐learn^29^ and statsmodel^30^ Python libraries. Longitudinal blood samples were only obtained for two of the five participants after long‐term treatment. Plasma pTau217 concentrations were determined using the S‐PLEX human pTau217 assay (MSD, Rockville, Maryland) and plasma protein concentrations using the BCA assay (Thermo Scientific, Waltham, MA).

## 3 RESULTS

At baseline, three of the participants, all female, presented with late‐onset AD (LOAD), and two participants, both male, with early‐onset AD (EOAD), defined as a diagnosis of AD before the age of 65. All five participants had mild AD at the start of the original trial (CONSORT diagram, Figure S1), with plasma pTau217 values above the cutoff considered diagnostic for AD^31^ (Table 1) and experienced only mild adverse events over the ∼30 months combined duration of the original trial and the open‐label extension periods. No adverse events were reported by participants on structured questionnaires at follow‐up visits in the open‐label extension period. At baseline, one participant using the control setting reported one episode of nervousness which resolved 10 min after initial presentation and another participant using the control device reported tiredness after 1 h of stimulation which eventually also resolved after 1 month of daily “control” stimulation.

**TABLE 1 alz70792-tbl-0001:** Baseline demographics of AD patients in long‐term extension

Characteristic	LOAD1	LOAD2	LOAD3	EOAD1	EOAD2
Years of age at baseline	76	88	81	63	55
Age of onset	72	87	79	61	54
Gender	F	F	F	M	M
Years of education	12	20	10	12	18
MMSE	22	26	22	24	23
MOCA	20	23	16	20	12
ADAS‐Cog	13	7	18	18	24
Global CDR	1	1	1	1	1
CDR SOB	6	6	5.5	5	4.5
FAS	10	20	19	10	13
APOE	E3/E3	E3/E3	E3/E3	E3/E3	E3/E4
Taking Aricept (n (%))	Yes	Yes	No	No	Yes
**Plasma pTau217 (pg/mL**)					
At baseline^a^	**27.6**	**9.78**	12.13	9.77	25.97
After ∼30 months of GENUS	**14.5**	**7.88**	ND	ND	ND

*Note*: Bolded values indicate pTau217 concentrations from participants with longitudinal data.

Abbreviations: AD, Alzheimer's disease; APOE, apolipoprotein E; CDR, Clinical Dementia Rating; CDR‐SOB, Clinical Dementia Rating Sum of Boxes; EOAD, early‐onset Alzheimer's disease; FAS, Functional Assessment Scale; GENUS, Gamma Entrainment Using Sensory; LOAD, late‐onset Alzheimer's disease; MMSE, Mini‐Mental State Examination; MOCA, Montreal Cognitive Assessment.

^a^ pTau217 concentrations > 6.8 pg/mL are considered diagnostic for Alzheimer's disease.

**FIGURE 1:**
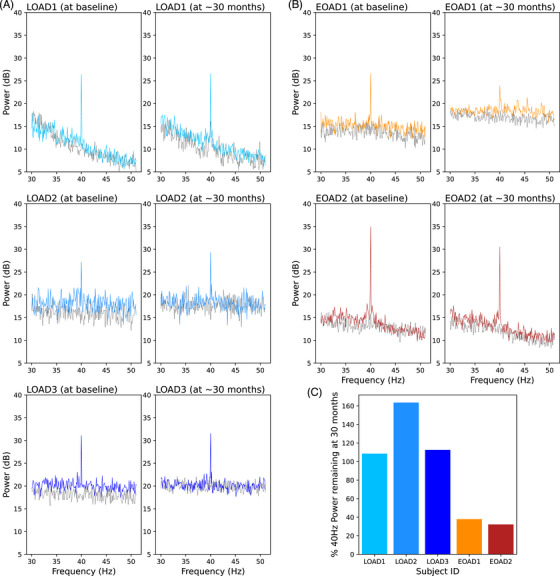
The 40 Hz power in response to GENUS. Power remains stable in late‐onset AD subjects (A, C), but declines in early‐onset AD subjects (B, C). EEG was first recorded for 1 min without stimulation (dotted black lines) and then for 1 min during GENUS stimulation (solid lines). AD, Alzheimer's disease; EEG, electroencephalography; GENUS, Gamma Entrainment Using Sensory Stimulation.

EEG at baseline indicated a robust power increase at 40 Hz in response to GENUS stimulation for all five participants (Figure 1). The EEG signal at 40 Hz was mostly located in the occipital regions, indicating a strong response to light stimulation (Figure S3). For one subject (LOAD2), 40 Hz signal was also detected in the frontal vertex regions, the expected location for response to sound stimulation.^32^ At ∼30 months, GENUS‐evoked 40 Hz power levels were increased over baseline for the three LOAD participants (109%, 164%, 113%, Figure 1A,C) but showed a steep decline for the two EOAD participants (38%, 32%, Figure 1B,C). Notably, the topographical pattern of the 40 Hz EEG signal remained the same for each subject (Figure S3). These data indicate that GENUS stimulation remained effective in the LOAD, but not in the EOAD participants.

**FIGURE 2:**
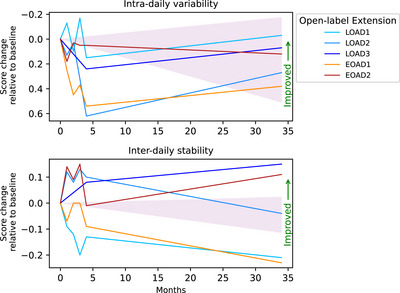
Changes in intra‐daily variability and inter‐daily stability in sleep over time. Shaded areas show expected ranges based on the standard deviations for patients with mild‐cognitive impairment given in Li et al., 2020.^33^ Y‐axis is reversed for intra‐daily variability.

Actigraphy measurements, conducted over a two‐week period at each time point, indicate that intra‐daily variability (i.e., fragmentation of rhythm) was reduced in four participants after starting 40 Hz GENUS and was in the range expected for participants with mild‐cognitive impairment^33^ for all five participants. Inter‐daily stability (i.e., strength of coupling to the external environment) improved for two of the five participants (Figure 2).

**FIGURE 3 alz70792-fig-0003:**
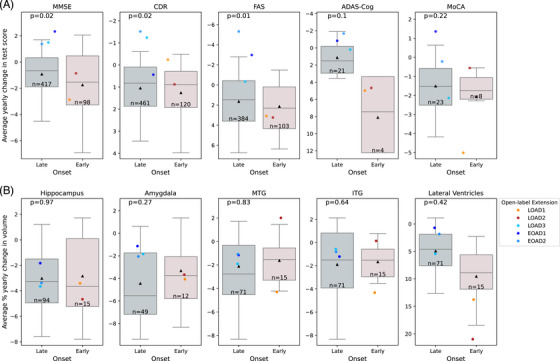
Overlay of open‐label extension participant data on control distributions of average yearly changes in test scores (A) or brain‐structure volumes (B). Changes in brain‐structure volume are given as a percentage of baseline volume. Boxes and whiskers show quartiles and 5th or 95th percentile, respectively, and black triangles show means for control subjects. Numbers of controls are given as “*n* = ”. *P*‐values were calculated based on the z‐scores of the average yearly changes for the three late‐onset subjects relative to a distribution of average yearly changes for 100,000 randomly selected samples of three control subjects. Y‐axes are reversed for CDR, FAS, ADAS‐Cog in (A) and for lateral ventricles in (B). For FAS, where not all of the 10 questions are applicable for all subjects, an average score was calculated by dividing the score sum by the number of questions answered. ADAS‐Cog, Alzheimer's Disease Assessment Scale‐Cognitive; CDR, Clinical Dementia Rating; FAS, Functional Assessment Scale; ITG, inferior temporal gyrus; MTG, middle temporal gyrus.

Cognitive testing included the five tests most commonly used in AD studies, namely the Mini‐Mental State Examination (MMSE)^34^, Clinical Dementia Rating Sum of Boxes (CDR‐SOB)^35^, Functional Assessment Scale (FAS)^36^, Montreal Cognitive Assessment (MoCA)^37^, and Alzheimer's Disease Assessment Scale‐Cognitive (ADAS‐Cog)^38^ tests. Control (i.e., no‐GENUS) data were obtained from the National Alzheimer's Coordinating Center (NACC), the Alzheimer's Disease Neuroimaging Initiative (ADNI), and the Longitudinal Early‐Onset Alzheimer's Disease Study (LEADS) databases (Table S1). From all three databases, we selected participants that were matched to open‐label extension participants on age range by sex and that had a CDR global score of 0.5 or 1 and an MMSE score ≥ 19 and ≤ 26 at baseline, matching the original inclusion criteria for the open‐label extension participants. We further filtered for control participants with test scores for at least three visits spaced out over at least 24 and at most 36 months, thus closely matching the testing window relative to baseline to that for the open‐label extension participants. Plotting test scores over time for each of the final control participants showed data from all three databases to be seamlessly integrated, without any discernible separation by database (Figure S4), validating their use as AD population controls. Using linear regression, we estimated an average yearly change in test scores for each participant and each test and corrected the score changes for the effect of years of education. Figure 3A shows the distribution of score changes for the control participants by test and sex/age‐of‐symptom onset as box plots and the corresponding score changes for the open‐label extension participants as dots. As expected, control subjects on average showed a score decline for all tests (Figure 3A, black triangles), which was more pronounced in EOAD than in LOAD controls. For the female LOAD participants, almost all scores showed less decline or more improvement than seen on average for control participants. For MMSE, CDR‐SOB, and FAS scores, this difference was significant when compared to a distribution of 100,000 randomly selected control samples (*p*‐values of 0.02, 0.02, and 0.01, respectively; Figure 3A and Figure S5). For the male EOAD participants, score changes fell as often below as above the average changes seen in controls. In this cohort, GENUS treatment was associated with stabilization or improvement in MMSE, CDR‐SOB, and FAS scores over a two‐year period in participants with LOAD but may be less effective in EOAD.

Structural MRI scans were used to measure volume changes in temporal lobe structures known to show atrophy with AD progression (hippocampus, amygdala, middle temporal gyrus – MTG, and inferior temporal gyrus – ITG) as well as ventricular dilation with AD progression.^39^, ^40^ No‐GENUS control data were again obtained from the NACC and ADNI databases. Control participants were matched to open‐label extension participants on sex, age range, MMSE score at baseline, and availability of at least two MRI scans spaced out over at least 11 and at most 36 months, and brain‐structure volumes were normalized to intracranial volume and corrected for the effect of baseline intracranial volume. Volume changes for the selected brain structures were not significantly different between open‐label extension and control participants but tended to be less severe in the LOAD participants (Figure 3B).

For two of the three LOAD participants, we were also able to evaluate changes in the plasma concentrations of phosphorylated tau 217 (pTau217), a highly sensitive biomarker for AD pathology that strongly correlates with amyloid burden.^41^, ^42^, ^43^ In both patients, we saw a decrease in plasma pTau217 levels after ∼2 years of daily GENUS stimulation, by 47% (LOAD1) and 19.4% (LOAD2); this decrease remained after normalizing to total plasma protein (54.9% and 19.2%). Notably, such a reduction in plasma pTau217 in response to a non‐invasive intervention has not previously been demonstrated in humans, and the pTau217 reduction observed here suggests that long‐term GENUS treatment may potentially reduce amyloid pathology, as has been shown in clinical trials of anti‐amyloid antibodies.^44^, [Fig alz70792-fig-0003]

## 4 DISCUSSION

This pilot study assessed the long‐term effects of daily 40 Hz multimodal GENUS in patients with mild AD. We found that daily 40 Hz audiovisual stimulation over 2 years is safe, feasible, and may slow cognitive decline and biomarker progression, especially in late‐onset AD patients.

The cognitive improvements observed in MMSE, CDR, and FAS scores among the LOAD participants in this cohort are noteworthy, as these measures are commonly used in clinical trials to assess global cognitive function, dementia severity, and everyday functional capacity. Our positive findings regarding FAS are consistent with results from Cimenser et al. (2021), who reported similar improvements in activities of daily living, as measured by the ADCS‐ADL scale, after 6 months of 40 Hz audiovisual stimulation. This convergence underscores the potential of GENUS to maintain daily functional abilities in patients with mild dementia due to AD. Interestingly, similar benefits were not seen in the MoCA nor the ADAS‐Cog scores of the LOAD patients. The reasons for this difference are not clear.

In the EOAD participants, no significant benefits were seen with any of the five cognitive tests. In these EOAD participants, EEG showed steeply reduced 40 Hz activity in response to GENUS at 30 months, while EEG responses in the LOAD participants were largely unchanged from baseline. These findings suggest that GENUS may be less effective in EOAD patients, potentially owing to broad pathological differences from LOAD that could contribute to differential responses.^45^ Future research should explore predictors of treatment response, such as genetic and pathological markers.

We did not observe significant differences in volume loss of temporal lobe structures between the open‐label extension and the control subjects. While both GENUS and repetitive transcranial magnetic stimulation have previously been reported to slow volume loss in brain structures known to be affected by AD,^22^, ^46^ these studies assessed volume loss over a window of only 3–6 months.

Additionally, circadian rhythmicity – documented using the actigraphy data – improved in some participants following 40 Hz GENUS, aligning with our earlier findings^22^ and those reported in Cimenser et al. (2021), highlighting the potential role for GENUS in stabilizing sleep and circadian patterns in AD patients.

Notably, one of the most compelling findings from this study was the significant reduction of plasma pTau217, a biomarker strongly correlated with AD pathology, in the two LOAD patients in whom follow‐up blood samples were available. These results suggest that GENUS could have direct biological impacts on AD pathology, warranting further mechanistic exploration in larger randomized trials.

This extension study followed an open‐label design and did not include any controls. It is noteworthy that offering the active treatment to all participants in the open‐label extension greatly increased their willingness to enroll in a randomized controlled trial with a placebo arm and to continue using the 40 Hz GENUS device over a period of years. Lack of a control arm was overcome by using data from the NACC, ADNI, and LEADS studies as controls. These nationwide studies are sufficiently large and diverse to allow matching of participants on sex, age, and cognitive status to the participants in our extension study. However, we cannot exclude a bias from patient self‐selection, and it remains unclear if the general population would be as diligent in their compliance with the use of the 40 Hz GENUS device as the participants in our extension study.

Another limitation is that sex and age of onset were confounded in our cohort (all LOAD participants were female; all EOAD participants were male). Given well‐established differences between EOAD and LOAD in brain network involvement and clinical phenotype, with EOAD showing a more aggressive disease trajectory,^47^, ^48^, ^49^ we emphasize age of onset as the more biologically meaningful distinction in this study.

Our findings extend previous shorter‐term studies, demonstrating the long‐term safety of GENUS and highlighting the feasibility of at‐home treatment. These results strengthen the rationale for broader implementation and evaluation of GENUS as a long‐term treatment modality. Future research should prioritize large‐scale long‐term follow‐up studies as well as shorter‐term randomized controlled trials to derive optimal treatment parameters and investigate the mechanistic underpinnings of GENUS.

## CONFLICT OF INTEREST STATEMENT

L.H.T. is a scientific co‐founder, SAB member, and member of the Board of Directors of Cognito Therapeutics. E.S.B. is a scientific co‐founder and SAB member of Cognito Therapeutics. E.B.K. has consulted for/received honoraria from the Sleep Research Society, the National Sleep Foundation, Circadian Therapeutics, and the Buck Institute on Aging; She has received travel/registration reimbursements from Lighten Up/EPFL Pavilions, the World Sleep Society, The Santa Fe Institute, the Society for Research in Biological Rhythms, the Sleep Research Society and the Lorentz Center; her partner owns Chronsulting. B.C.D. is a consultant for Acadia, Alector, Arkuda, Biogen, Denali, Lilly, Merck, Novartis, Takeda, and Wave Lifesciences, and receives royalties from Cambridge University Press, Elsevier, and Oxford University Press. D.C., G.D.W., B.J., H.J.S., N.P.M., E.K., V.S.F.A., M.Q., K.A., E.R., A.B., M.Z., R.P., R.F., U.G., B.H., P.K., S.A., and E.N.B. have nothing to disclose. Author disclosures are available in the Supporting Information.

## CONSENT STATEMENT

This study was registered on ClinicalTrials.gov (NCT04055376) and received ethical approval from the Committee on the Use of Humans as Experimental Subjects (COUHES) at the Massachusetts Institute of Technology. Prior to participation, all individuals provided written informed consent. All procedures were conducted in accordance with ethical standards of the institutional and/or national research committee and with the 2013 revision of the Declaration of Helsinki.

## Supporting information



Supporting information

Supporting information

Supporting information

Supporting information

Supporting information

Supporting information

Supporting information

## References

[1] Canter RG , Penney J , Tsai L‐H . The road to restoring neural circuits for the treatment of Alzheimer's disease. Nature. 2016;539:187‐196. https://pubmed.ncbi.nlm.nih.gov/27830780/ 27830780 10.1038/nature20412

[2] Uhlhaas PJ , Singer W . Neural synchrony in brain disorders: relevance for cognitive dysfunctions and pathophysiology. Neuron. 2006;52:155‐168. https://pubmed.ncbi.nlm.nih.gov/17015233/ 17015233 10.1016/j.neuron.2006.09.020

[3] Nimmrich V , Draguhn A , Axmacher N . Neuronal network oscillations in neurodegenerative diseases. Neuromolecular Med. 2015;17:270‐284. https://pubmed.ncbi.nlm.nih.gov/25920466/ 25920466 10.1007/s12017-015-8355-9

[4] Herrmann CS , Demiralp T . Human EEG gamma oscillations in neuropsychiatric disorders. Clin Neurophysiol. 2005;116:2719‐2733. https://pubmed.ncbi.nlm.nih.gov/16253555/ 16253555 10.1016/j.clinph.2005.07.007

[5] Palop JJ , Mucke L . Network abnormalities and interneuron dysfunction in Alzheimer disease. Nat Rev Neurosci. 2016;17:777‐792. https://pubmed.ncbi.nlm.nih.gov/27829687/ 27829687 10.1038/nrn.2016.141 PMC8162106

[6] van Deursen JA , Vuurman EFPM , Verhey FRJ , van Kranen‐Mastenbroek VHJM , Riedel WJ . Increased EEG gamma band activity in Alzheimer's disease and mild cognitive impairment. J Neural Transm (Vienna). 2008;115:1301‐1311. https://pubmed.ncbi.nlm.nih.gov/18607528/ 18607528 10.1007/s00702-008-0083-y PMC2525849

[7] Stam CJ , van Cappellen van Walsum AM , Pijnenburg YAL , et al. Generalized synchronization of MEG recordings in Alzheimer's Disease: evidence for involvement of the gamma band. J Clin Neurophysiol. 2002;19:562‐574. https://pubmed.ncbi.nlm.nih.gov/12488788/ 12488788 10.1097/00004691-200212000-00010

[8] Adaikkan C , Tsai L‐H . Gamma entrainment: impact on neurocircuits, Glia, and therapeutic opportunities. Trends Neurosci. 2020;43:24‐41. https://pubmed.ncbi.nlm.nih.gov/31836315/ 31836315 10.1016/j.tins.2019.11.001

[9] Iaccarino HF , Singer AC , Martorell AJ , et al. Gamma frequency entrainment attenuates amyloid load and modifies microglia. Nature. 2016;540:230‐235. https://pubmed.ncbi.nlm.nih.gov/27929004/ 27929004 10.1038/nature20587 PMC5656389

[10] Adaikkan C , Middleton SJ , Marco A , et al. Gamma entrainment binds higher‐order brain regions and offers neuroprotection. Neuron. 2019;102:929‐943. https://pubmed.ncbi.nlm.nih.gov/31076275/. e8. 31076275 10.1016/j.neuron.2019.04.011 PMC6697125

[11] Martorell AJ , Paulson AL , Suk H‐J , et al. Multi‐sensory gamma stimulation ameliorates Alzheimer's‐associated pathology and improves cognition. Cell. 2019;177:256‐271. https://pubmed.ncbi.nlm.nih.gov/30879788/. e22. 30879788 10.1016/j.cell.2019.02.014 PMC6774262

[12] Murdock MH , Yang C‐Y , Sun N , et al. Multisensory gamma stimulation promotes glymphatic clearance of amyloid. Nature. 2024;627:149‐156. https://pubmed.ncbi.nlm.nih.gov/38418876/ 38418876 10.1038/s41586-024-07132-6 PMC10917684

[13] Kim S‐H , Park S‐S , Kim C‐J , Kim T‐W . Exercise with 40‐Hz light flicker improves hippocampal insulin signaling in Alzheimer disease mice. J Exerc Rehabil. 2022;18:20‐27. https://pubmed.ncbi.nlm.nih.gov/35356135/ 35356135 10.12965/jer.2244042.021 PMC8934612

[14] Shen Q , Wu X , Zhang Z , Zhang D , Yang S , Xing D . Gamma frequency light flicker regulates amyloid precursor protein trafficking for reducing β‐amyloid load in Alzheimer's disease model. Aging Cell. 2022;21:e13573. https://pubmed.ncbi.nlm.nih.gov/35199454/ 35199454 10.1111/acel.13573 PMC8920449

[15] Yao Y , Ying Y , Deng Q , et al. Non‐invasive 40‐Hz light flicker ameliorates Alzheimer's‐associated rhythm disorder via regulating central circadian clock in mice. Front Physiol. 2020;11:294. https://pubmed.ncbi.nlm.nih.gov/32390857/ 32390857 10.3389/fphys.2020.00294 PMC7193101

[16] Zheng L , Yu M , Lin R , et al. Rhythmic light flicker rescues hippocampal low gamma and protects ischemic neurons by enhancing presynaptic plasticity. Nat Commun. 2020;11:3012. 10.1038/s41467-020-16826-0 32541656 PMC7296037

[17] Tian T , Qin X , Wang Y , Shi Y , Yang X . 40 Hz light flicker promotes learning and memory via long term depression in wild‐type mice. J Alzheimers Dis. 2021;84:983‐993. 10.3233/JAD-215212 34602491

[18] Park S‐S , Park H‐S , Kim C‐J , et al. Combined effects of aerobic exercise and 40‐Hz light flicker exposure on early cognitive impairments in Alzheimer's disease of 3×Tg mice. J Appl Physiol. 2022;132:1054‐1068. 10.1152/japplphysiol.00751.2021 35201933

[19] Park S‐S , Park H‐S , Kim C‐J , et al. Physical exercise during exposure to 40‐Hz light flicker improves cognitive functions in the 3xTg mouse model of Alzheimer's disease. Alzheimers Res Ther. 2020;12:62. 10.1186/s13195-020-00631-4 32434556 PMC7240923

[20] Nazari M , Vajed‐Samiei T , Torabi N , et al. The 40‐Hz white light‐emitting diode (LED) improves the structure‐function of the brain mitochondrial KATP channel and respiratory chain activities in amyloid beta toxicity. Mol Neurobiol. 2022;59:2424‐2440. 10.1007/s12035-021-02681-7 35083663

[21] Wang W , Zhang X , He R , Li S , Fang D , Pang C . Gamma frequency entrainment rescues cognitive impairment by decreasing postsynaptic transmission after traumatic brain injury. CNS Neurosci Ther. 2023;29:1142‐1153. 10.1111/cns.14096 36740277 PMC10018095

[22] Chan D , Suk H‐J , Jackson BL , et al. Gamma frequency sensory stimulation in mild probable Alzheimer's dementia patients: results of feasibility and pilot studies. PLoS One. 2022;17:e0278412. https://pubmed.ncbi.nlm.nih.gov/36454969/ 36454969 10.1371/journal.pone.0278412 PMC9714926

[23] He Q , Colon‐Motas KM , Pybus AF , et al. A feasibility trial of gamma sensory flicker for patients with prodromal Alzheimer's disease. Alzheimers Dement (N Y). 2021;7:e12178. https://pubmed.ncbi.nlm.nih.gov/34027028/ 34027028 10.1002/trc2.12178 PMC8118113

[24] Cimenser A , Hempel E , Travers T , et al. Sensory‐evoked 40‐Hz gamma oscillation improves sleep and Daily Living activities in Alzheimer's disease patients. Front Syst Neurosci. 2021;15:746859. https://pubmed.ncbi.nlm.nih.gov/34630050/ 34630050 10.3389/fnsys.2021.746859 PMC8500065

[25] Da X , Hempel E , Brickman AM , Hajós M , Kern R , Cimenser A . Spectris™ treatment preserves corpus callosum structure in Alzheimer's disease. Front Neurol [Internet]. 2024;15:1452930. https://pubmed.ncbi.nlm.nih.gov/39479005/ 39479005 10.3389/fneur.2024.1452930 PMC11522122

[26] Da X , Hempel E , Ou Y , et al. Noninvasive gamma sensory stimulation may reduce white matter and myelin loss in Alzheimer's disease. J Alzheimers Dis. 2024;97:359‐372. https://pubmed.ncbi.nlm.nih.gov/38073386/ 38073386 10.3233/JAD-230506 PMC10789351

[27] Hajós M , Boasso A , Hempel E , et al. Safety, tolerability, and efficacy estimate of evoked gamma oscillation in mild to moderate Alzheimer's disease. Front Neurol. 2024;15:1343588. https://pubmed.ncbi.nlm.nih.gov/38515445/ 38515445 10.3389/fneur.2024.1343588 PMC10957179

[28] Gramfort A , Luessi M , Larson E , et al. MEG and EEG data analysis with MNE‐Python. Front Neurosci. 2013;7:267. 10.3389/fnins.2013.00267 24431986 PMC3872725

[29] Pedregosa F , Varoquaux G , Gramfort A , et al. Scikit‐learn: machine learning in python. *arXiv*. 2012. http://arxiv.org/abs/1201.0490 10.3389/fninf.2014.00014 PMC3930868 24600388

[30] Seabold S , Perktold J , Statsmodels: econometric and statistical modeling with python. Proceedings of the Python in Science Conference. SciPy 2010; p. 92‐96. https://doi.curvenote.com/10.25080/Majora‐92bf1922‐011

[31] FDA Clears First Blood Test Used in Diagnosing Alzheimer's Disease [Internet]. fda.gov. 2025. https://www.fda.gov/news‐events/press‐announcements/fda‐clears‐first‐blood‐test‐used‐diagnosing‐alzheimers‐disease

[32] Wolpaw JR , Penry JK . A temporal component of the auditory evoked response. Electroencephalogr Clin Neurophysiol. 1975;39:609‐620. doi:10.1016/0013-4694(75)90073-5 53139

[33] Li P , Gao L , Gaba A , et al. Circadian disturbances in Alzheimer's disease progression: a prospective observational cohort study of community‐based older adults. Lancet Healthy Longev. 2020;1:e96‐105. 10.1016/s2666-7568(20)30015-5 34179863 PMC8232345

[34] Folstein MF , Folstein SE , McHugh PR . “Mini‐mental state”. A practical method for grading the cognitive state of patients for the clinician. J Psychiatr Res. 1975;12:189‐198. 10.1016/0022-3956(75)90026-6 1202204

[35] Morris JC . The Clinical Dementia Rating (CDR): current version and scoring rules. Neurology. 1993;43:2412‐2414. 10.1212/wnl.43.11.2412-a 8232972

[36] Pfeffer RI , Kurosaki TT , Harrah CH, Jr , Chance JM , Filos S . Measurement of functional activities in older adults in the community. J Gerontol. 1982;37:323‐329. 10.1093/geronj/37.3.323 7069156

[37] Nasreddine ZS , Phillips NA , Bédirian V , et al. The Montreal Cognitive Assessment, MoCA: a brief screening tool for mild cognitive impairment. J Am Geriatr Soc. 2005;53:695‐699. 10.1111/j.1532-5415.2005.53221.x 15817019

[38] Rosen WG , Mohs RC , Davis KL . A new rating scale for Alzheimer's disease. Am J Psychiatry. 1984;141:1356‐1364. 10.1176/ajp.141.11.1356 6496779

[39] Schuff N , Woerner N , Boreta L , et al. MRI of hippocampal volume loss in early Alzheimer's disease in relation to ApoE genotype and biomarkers. Brain. 2009;132:1067‐1077. 10.1093/brain/awp007 19251758 PMC2668943

[40] Risacher SL , Shen L , West JD , et al. Longitudinal MRI atrophy biomarkers: relationship to conversion in the ADNI cohort. Neurobiol Aging. 2010;31:1401‐1418. 10.1016/j.neurobiolaging.2010.04.029 20620664 PMC2904350

[41] Schindler SE , Petersen KK , Saef B , et al. Head‐to‐head comparison of leading blood tests for Alzheimer's disease pathology. Alzheimers Dement. 2024;20:8074‐8096. https://pubmed.ncbi.nlm.nih.gov/39394841/ 39394841 10.1002/alz.14315 PMC11567821

[42] Therriault J , Vermeiren M , Servaes S , et al. Association of phosphorylated tau biomarkers with amyloid positron emission tomography vs. tau positron emission tomography. JAMA Neurol. 2023;80:188‐199. https://pubmed.ncbi.nlm.nih.gov/36508198/ 36508198 10.1001/jamaneurol.2022.4485 PMC9856704

[43] Rissman RA , Langford O , Raman R , et al. Plasma Aβ42/Aβ40 and phospho‐tau217 concentration ratios increase the accuracy of amyloid PET classification in preclinical Alzheimer's disease. Alzheimers Dement. 2024;20:1214‐1224. https://pubmed.ncbi.nlm.nih.gov/37932961/ 37932961 10.1002/alz.13542 PMC10916957

[44] Sims JR , Zimmer JA , Evans CD , et al. Donanemab in Early Symptomatic Alzheimer Disease: the TRAILBLAZER‐ALZ 2 randomized clinical trial. JAMA. 2023;330:512‐527. doi:10.1001/jama.2023.13239 37459141 PMC10352931

[45] Chiaravalloti A , Koch G , Toniolo S , et al. Comparison between early‐onset and late‐onset Alzheimer's disease patients with amnestic presentation: CSF and (18)F‐FDG PET Study. Dement Geriatr Cogn Dis Extra. 2016;6:108‐119. doi:10.1159/000441776 27195000 PMC4868930

[46] Mencarelli L , Torso M , Borghi I , et al. Macro and micro structural preservation of grey matter integrity after 24 weeks of rTMS in Alzheimer's disease patients: a pilot study. Alz Res Therapy. 2024;16:152. doi:10.1186/s13195-024-01501-z PMC11225141 38970141

[47] Reitz C , Rogaeva E . Beecham GW Late‐onset vs. nonmendelian early‐onset Alzheimer disease: a distinction without a difference?. Neurol Genet. 2020;6:e512. doi:10.1212/NXG.0000000000000512 33225065 PMC7673282

[48] Tort‐Merino A , Falgàs N , Allen IE , et al. Early‐onset Alzheimer's disease shows a distinct neuropsychological profile and more aggressive trajectories of cognitive decline than late‐onset. Ann Clin Transl Neurol. 2022;9:1962‐1973. doi:10.1002/acn3.51689 36398437 PMC9735361

[49] Wattmo C , Wallin ÅK . Early‐ versus late‐onset Alzheimer's disease in clinical practice: cognitive and global outcomes over 3 years. Alzheimers Res Ther. 2017;9:70. doi:10.1186/s13195-017-0294-2 28859660 PMC5580278

