# Supplementary figures and images for: Gamma sensory stimulation in mild Alzheimer's dementia: An open‐label extension study
Source: Alzheimers Dement. 2025 Oct 25;21(10):e70792. doi: 10.1002/alz.70792 (PMC12552893; doi:10.1002/alz.70792)

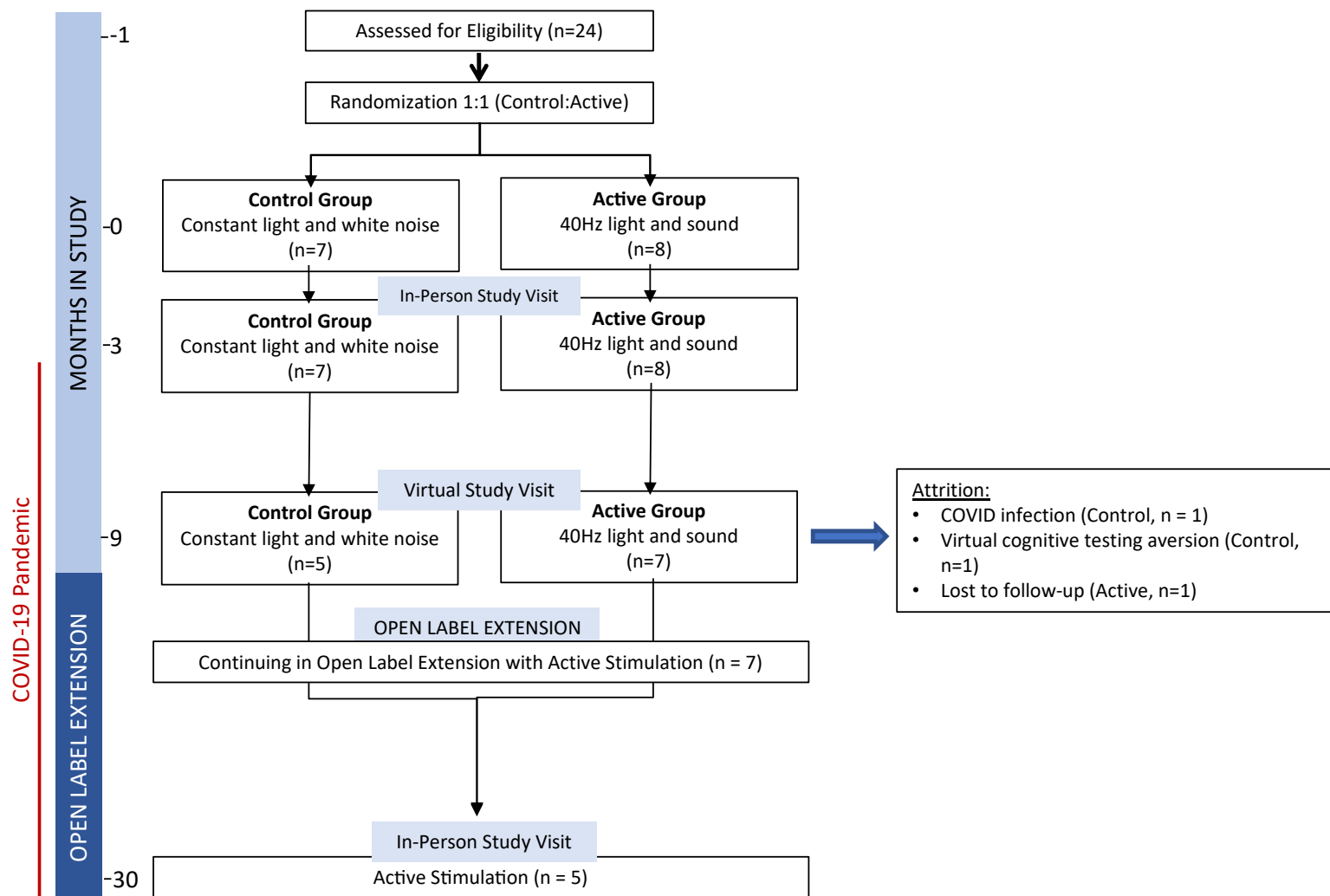

## Supplemental Figure 1. CONSORT diagram

Adapted from Chan et al. (2022), PLOS ONE 17(12): e0278412. DOI: 10.1371/journal.pone.0278412.

Supplement: Supplementary file 1 — Supporting information [file ALZ-21-e70792-s002.pdf]
