# Supplementary material for: Gamma sensory stimulation in mild Alzheimer's dementia: An open‐label extension study
Source: Alzheimers Dement. 2025 Oct 25;21(10):e70792. doi: 10.1002/alz.70792 (PMC12552893; doi:10.1002/alz.70792)
Supplement: Supplementary file 2 — Supporting information [file ALZ-21-e70792-s006.pdf]

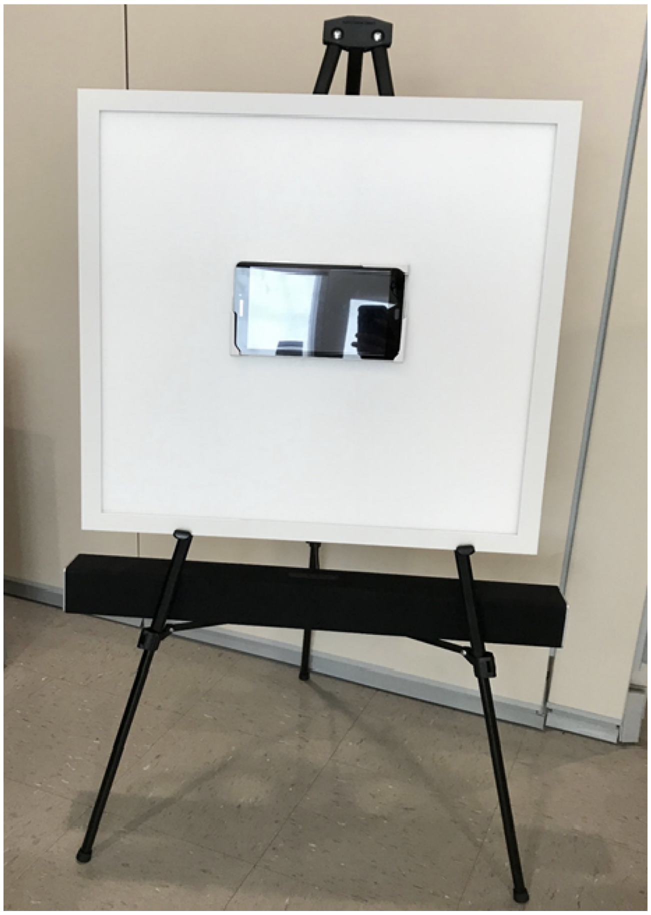

Supplemental Figure 2. GENUS Device

The device consists of a 2 feet × 2 feet programmable LED light panel (white board), with a tablet mounted at its center to maintain visual engagement. A soundbar (black bar) is positioned below the panel to deliver synchronized auditory stimulation. The setup is supported by a black easel and configured such that the tablet is at eye level to deliver entertainment when the participant is seated approximately 5 feet away.

Adapted from Chan et al (2022). PLOS ONE 17(12): e0278412. DOI: 10.1371/journal.pone.0278412.
