# Supplementary material for: Gamma sensory stimulation in mild Alzheimer's dementia: An open‐label extension study
Source: Alzheimers Dement. 2025 Oct 25;21(10):e70792. doi: 10.1002/alz.70792 (PMC12552893; doi:10.1002/alz.70792)
Supplement: Supplementary file 3 — Supporting information [file ALZ-21-e70792-s007.pdf]

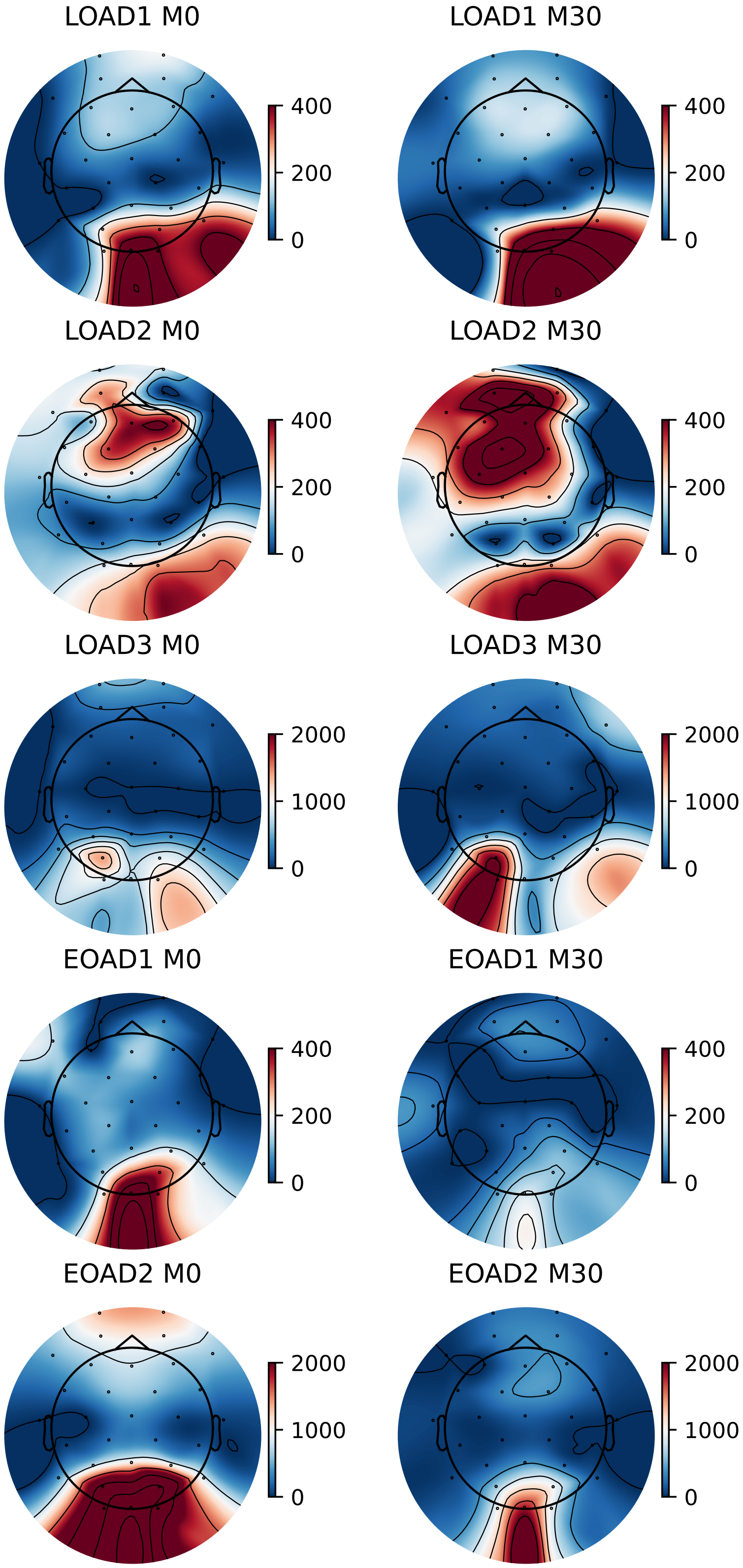

Supplemental Figure 3. Topographic maps of 40Hz power during light and sound stimulation. Maps showing distribution of 32 EEG leads from baseline and month 30 recordings. (EOAD, early-onset AD; LOAD, late-onset AD; M0, baseline; M30, month 30).
