# Supplementary material for: Gamma sensory stimulation in mild Alzheimer's dementia: An open‐label extension study
Source: Alzheimers Dement. 2025 Oct 25;21(10):e70792. doi: 10.1002/alz.70792 (PMC12552893; doi:10.1002/alz.70792)
Supplement: Supplementary file 4 — Supporting information [file ALZ-21-e70792-s005.pdf]

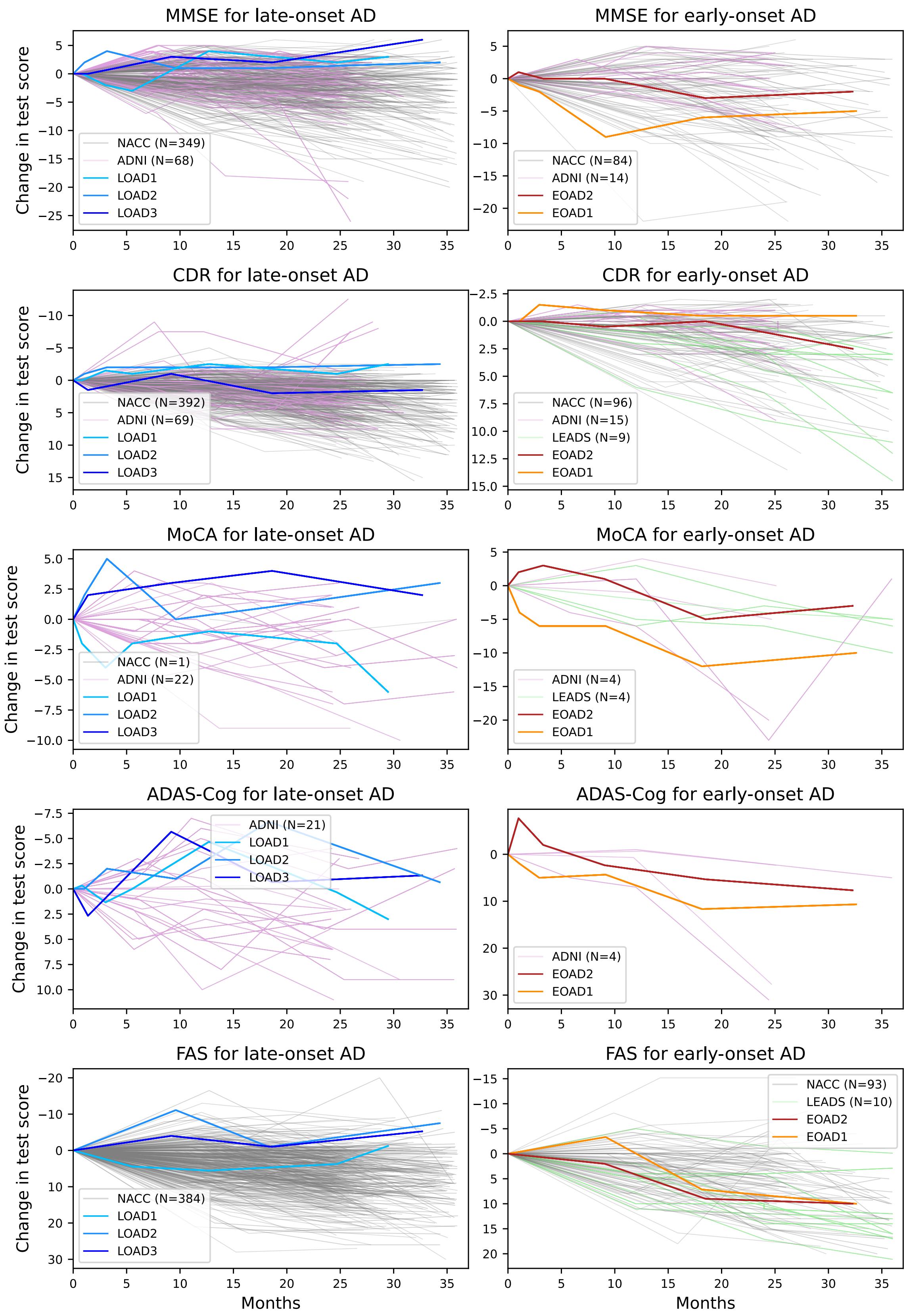

Supplemental Figure 4. Changes in test scores over time. Each line shows test score changes for one subject, with line color indicating study. “N=”: number of control subjects from each study.
