# Supplementary material for: Gamma sensory stimulation in mild Alzheimer's dementia: An open‐label extension study
Source: Alzheimers Dement. 2025 Oct 25;21(10):e70792. doi: 10.1002/alz.70792 (PMC12552893; doi:10.1002/alz.70792)
Supplement: Supplementary file 5 — Supporting information [file ALZ-21-e70792-s004.pdf]

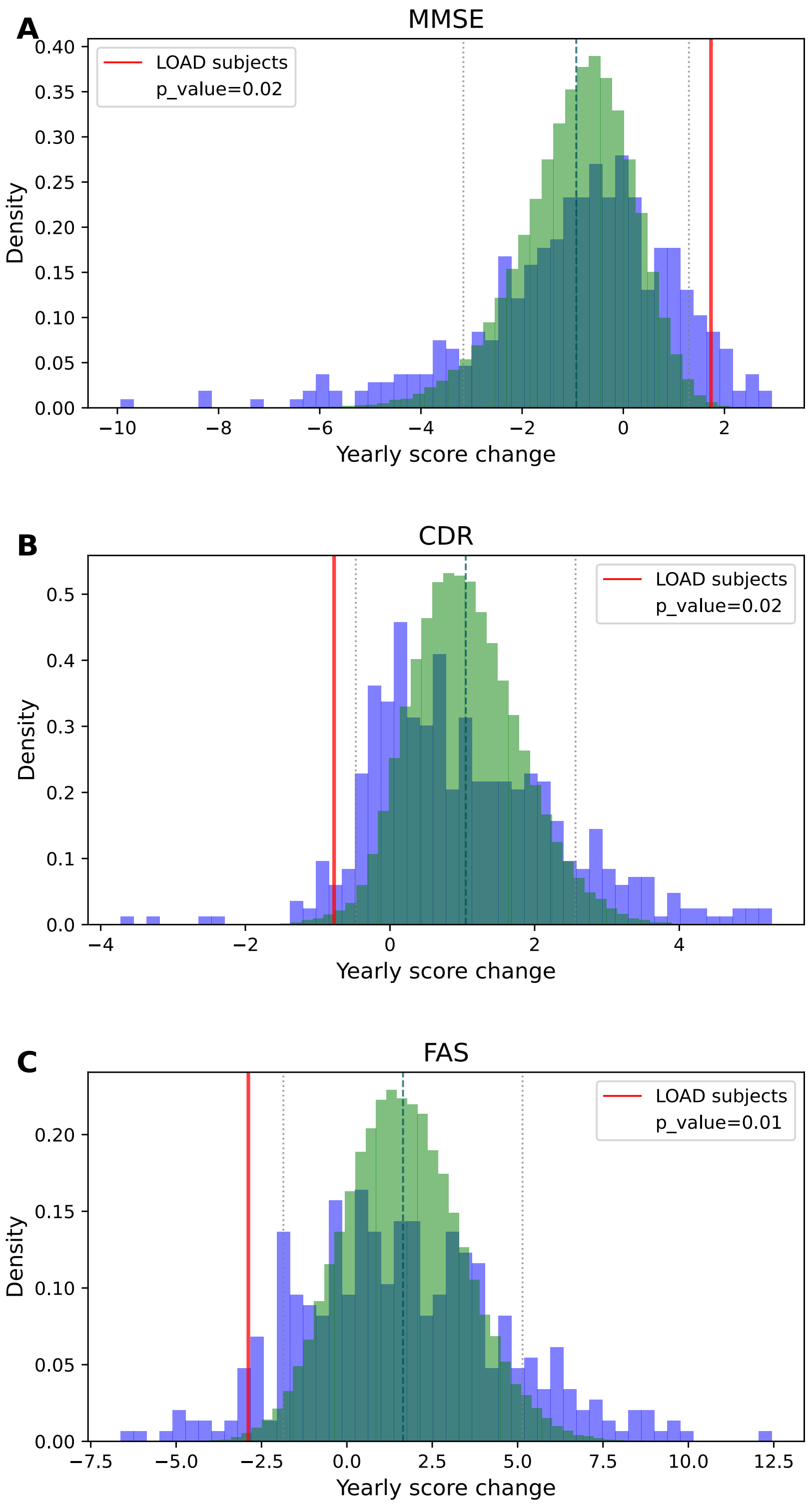

Supplemental Figure 5. Distributions of average yearly MMSE, CDR, and FAS score changes for late-onset AD subjects. Green: observed score changes for control subjects; blue: mean score changes for 100,000 random samples of observed score changes for three control subjects; dashed line: overlapping means of observed and bootstrapped distributions; dotted lines: two standard deviations from mean for bootstrapped distribution; red line: mean score change for LOAD1, LOAD2, and LOAD3. P-values are based on z-score of LOAD1,2,3 mean relative to bootstrapped distribution.
