# Supplementary material for: Gamma sensory stimulation in mild Alzheimer's dementia: An open‐label extension study
Source: Alzheimers Dement. 2025 Oct 25;21(10):e70792. doi: 10.1002/alz.70792 (PMC12552893; doi:10.1002/alz.70792)
Supplement: Supplementary file 6 — Supporting information [file ALZ-21-e70792-s001.docx]

| **Supplemental Table 1:** Datasets for control subjects | | |
| --- | --- | --- |
|  |  |  |
| **Study** | **Data Type** | **Source** |
| NACC | Demographic data | https://naccdata.org |
| NACC | Cognitive testing data | https://naccdata.org |
| NACC | MRI data | https://naccdata.org |
| ADNI1,2,3,GO | Demographic data | https://adni.loni.usc.edu/ |
| ADNI1,2,3,GO | Cognitive testing data | https://adni.loni.usc.edu/ |
| ADNI3 | MRI data | https://adni.loni.usc.edu/ |
| ADNI1,2,GO | MRI data | Ledig et al, 2018, *Scientific Reports* 8: 11258. |
| LEADS | Demographic data | https://leads-study.medicine.iu.edu/ |
| LEADS | Cognitive testing data | https://leads-study.medicine.iu.edu/ |
